# Interaction of lncRNA Gm2044 and EEF2 promotes estradiol synthesis in ovarian follicular granulosa cells

**DOI:** 10.1186/s13048-023-01232-z

**Published:** 2023-08-23

**Authors:** Ke Hu, Chen Wang, Yifan Xu, Fan Li, Xuefeng Han, Chuanwang Song, Meng Liang

**Affiliations:** 1 School of Life Science, Bengbu Medical College, Bengbu, China; 2 School of Laboratory Medicine, Bengbu Medical College, Bengbu, China

**Keywords:** lncRNA, Gm2044, EEF2, Estradiol, Granulosa cells

## Abstract

The functions and molecular mechanisms of long noncoding RNA (lncRNA) in reproduction have been widely studied at present. However, lncRNA regulating hormone synthesis in ovarian follicular granulosa cells has not been sufficiently studied. Our previous research demonstrated that lncRNA Gm2044 could promote estradiol synthesis in follicular granulosa cells. In this study, we identified 21 binding proteins of lncRNA Gm2044 in ovarian follicles using comprehensive identification of RNA-binding proteins by mass spectrometry (ChIRP-MS). RNA immunoprecipitation (RNA IP) and reverse transcription PCR (RT-PCR) confirmed that lncRNA Gm2044 can interact with eukaryotic translation elongation factor 2 (EEF2) protein. Furthermore, we constructed lncRNA Gm2044 knockout mice using the CRISPR/Cas9 method. Although the follicular development and fertility of female lncRNA Gm2044 knockout mice were not affected, the serum estradiol concentration in female lncRNA Gm2044 knockout mice significantly decreased. Western blotting and ELISA revealed that lncRNA Gm2044 may promote the binding of EEF2 to *Nr5a1* mRNA and then enhance the *Nr5a1* mRNA translation, and the upregulated NR5A1 protein can strengthen estradiol synthesis. To determine the potential signaling pathway of lncRNA Gm2044 regulating estradiol synthesis, transcriptome sequencing was performed for ovaries of adult lncRNA Gm2044 knockout mice, which identified 565 significant up-regulated genes and 303 significant down-regulated genes, which were then analyzed with Gene Ontology (GO) and the Kyoto Encyclopedia of Genes and Genomes (KEGG) and validated by molecular experiments. Understanding how lncRNA Gm2044/EEF2 protein regulates estradiol synthesis will help treat estrogen-related reproductive diseases.

**Supplementary Information:**

The online version contains supplementary material available at 10.1186/s13048-023-01232-z.

## Introduction

The functions and molecular mechanisms of lncRNA in female reproduction have been reported at present [1, 2]. Decreased expression of lncRNA TINCR in the placentas of pre-eclampsia patients can enhance cellular proliferation, migration, and invasion by promoting the Wnt/β-catenin signaling pathway [1]. LncRNA AFAP1-AS1 coordinates with EZH2 to repress *Dusp5* transcription by modifying H3K27m3 of the *Dusp5* promoter and then promotes proliferation, migration, and invasion of trophoblast cells [2]. Recurrent abortion is associated with high expression of lncRNA GAS5, which keeps the trophoblast Th1 bias by serving as competing endogenous RNA for miR-140-5p [3]. Increased lncRNA LINC01088 suppresses proliferation and enhances apoptosis for trophoblast cells by affecting JNK and p38 MAPK signaling in recurrent abortion [4]. In addition, upregulated lncRNA NEAT1 in the ovaries of patients and rats with polycystic ovary syndrome may inhibit sex hormone synthesis, aggravate pathological change, and promote granulosa cell apoptosis through regulating miR-381 and IGF [5]. The in-depth molecular mechanism of lncRNA regulation of female fertility needs to be further investigated.

The coordination of lncRNA and protein has been proven to be involved in the pathological and physiological processes of mammals [6–8]. Increased CREB protein in diabetic nephropathy induces renal inflammation and ruins podocytes via regulating lncRNA DLX6-AS1 [6]. LncRNA ZFAS1 interacts with PABP2 protein and then binds to Srebp1 mRNA, which promotes SREBP1 protein expression and lipogenesis to accelerate the development of colorectal cancer [9]. LncRNA SNHG16 can bind to CELF2 protein and promote CELF2 mRNA degradation, which enhances cellular proliferation and migration of acute myeloid leukemia by regulating PTEN signaling [7]. Increased lncRNA AL355338 accelerates aerobic glycolysis via binding ENO1 protein and affecting EGFR signaling in non-small-cell lung cancer [10]. In addition, lncRNA H19 is capable of influencing SNORA7A and affecting the process of DNA damage in osteosarcomas of Li-Fraumeni syndrome [8]. Researchers are paying more attention to the study that lncRNA and protein jointly affect life activities.

LncRNA can regulate hormone synthesis and secretion or mediate hormone effects in physiological and pathological processes [11–13]. LncRNA MALAT1 enhances the levels of estradiol and progesterone by affecting the miR-205/CREB1 signaling pathway and then inhibits apoptosis for mouse ovarian granulosa cells [11]. Follicle-stimulating hormone (FSH) production can be upregulated by lncRNA m433s1, which regulates FSH-β level by acting as competing endogenous RNA for miR-433 [12]. Estradiol promotes MCF-7 cell proliferation via upregulating lncRNA H19 expression in a time- and dose-dependent manner [13]. A high level of lncRNA TUG1 in pancreatic β cells can promote insulin secretion and inhibit cellular apoptosis [14]. The expression of lncRNA HOTAIR can be facilitated by estradiol and enhance cellular proliferation in breast cancer cells [15]. How lncRNA interacts with protein to regulate hormone production has not been well studied at present.

In this research, we identified the binding proteins of lncRNA Gm2044 by ChIRP-MS and confirmed the interaction through RNA IP and RT-PCR. Furthermore, lncRNA Gm2044 knockout mice were constructed using the CRISPR/Cas9 method and subjected to analysis of hormone secretion and reproductive phenotype. In addition, transcriptome sequencing was performed for ovaries of lncRNA Gm2044 knockout mice and validated by quantitative PCR (qPCR). Understanding lncRNA Gm2044/protein regulating hormone synthesis will be helpful for the treatment of hormone-related reproductive diseases.

## Materials and methods

### LncRNA Gm2044 knockout mice construction, plasmid and siRNA

CRISPR/Cas9 technology was used to introduce lncRNA Gm2044 mutation by non-homologous recombination repair, which resulted in the loss of lncRNA Gm2044 genomic sequence and function. Briefly, Cas9 mRNA and gRNA were acquired by in vitro transcription. Cas9 mRNA and gRNA were microinjected into the fertilized eggs of C57BL/6J mice to get F0 generation mice which were identified by genotyping and sequencing. The positive F0 mice were mated with C57BL/6J mice to obtain positive offspring mice. The weights of ovaries and mouse bodies for lncRNA Gm2044 knockout mice were statistically analyzed. The ovarian follicular development of lncRNA Gm2044 knockout mice was detected by hematoxylin-eosin staining (H&E, Servicebio, Wuhan, China). The gRNA sequences and primers for genotyping were listed in Table S1 and Table S2, respectively.

The plasmid for overexpression of lncRNA Gm2044 was described in our previous research [16]. The siRNA for mouse EEF2 (si-EEF2) and siRNA for negatice control (si-NC) were purchased from Shanghai GenePharma (Shanghai, China).

### ChIRP-MS and RNA IP

For ChIRP-MS, the ovarian cell suspension was reversibly cross-linked in situ with formaldehyde (Sangon Biotech, Shanghai, China), followed by cleaving with lysis buffer (50 mM Tris-HCl pH 7.0 (Sigma-Aldrich, St. Louis, MO, USA), 1% SDS (Sangon Biotech), 10 mM EDTA (Sigma-Aldrich), 1 mM PMSF (Sangon Biotech)) and hybridization with biotin-labeled RNA probes (control probe and Gm2044 probe). After the non-specific binding proteins were washed out under strong denaturation conditions, the obtained RNA-binding proteins were identified and quantitatively analyzed by liquid chromatography-tandem MS. Then, the interactive protein profile of lncRNA Gm2044 was constructed and analyzed with gene ontology (GO, http://geneontology.org/), the Kyoto Encyclopedia of Genes and Genomes (KEGG, https://www.genome.jp/kegg/) and STRING (https://cn.string-db.org/). The probes of lncRNA Gm2044 were listed in Table S3, and the control probe was described in a previous study [17]. For RNA IP, the corresponding RNA-protein complexes were precipitated using antibodies against EEF2 protein (ABclonal Technology, Wuhan, China), and the purified protein and RNA were then analyzed according to the protocol in previous studies [18, 19].

### RT-PCR and qPCR

Total RNA was extracted from tissues or cells using Trizol (Invitrogen, Carlsbad, CA, USA) and then reverse transcribed into cDNA using RT reagent (Accurate Biotechnology, Changsha, China). For qPCR, cDNA was used to analyze lncRNA Gm2044 and *Nr5a1* mRNA expression using the SYBR Green kit (Accurate Biotechnology). Primers for lncRNA Gm2044 of qPCR are listed in Table S4 and primers for β-Actin (reference gene) and *Nr5a1* of qPCR were described in a previous study [20]. For RT-PCR, cDNA was used to analyze lncRNA Gm2044 expression using a PCR kit (Beyotime Biotechnology, Shanghai, China). Primers for lncRNA Gm2044 and *Nr5a1* of RT-PCR were listed in Table S5.

### Transcriptome analysis

Ovaries of adult lncRNA Gm2044 heterozygous and knockout mice were isolated and then TRIzol was used to extract RNA from ovaries according to manufacturer’s instruction. The quality and concentration of RNA were analyzed by automatic nucleic acid detection and fluorogenic quantitative detection. Then, the library was built and sequenced, and the transcriptional abundance and expression levels were calculated according to our previous research [21]. GO (GO, http://geneontology.org/) and KEGG (https://www.genome.jp/kegg/) were used to analyze the downstream signaling pathway of lncRNA Gm2044 in ovaries.

### Western blotting and enzyme-linked immunosorbent assay (ELISA)

For western blotting, the protein was isolated from tissues or cells using RIPA reagent (Millipore, Bedford, MA, USA) supplemented with protease inhibitor (Roche, Mannheim, Germany), subjected to electrophoresis and membrane transfer, and then detected with antibodies of EEF2 (ABclonal Technology), NR5A1 (Invitrogen) and β-Actin (ABclonal Technology).

For ELISA, the concentration of estradiol in serum or cell culture medium was examined using the Mouse Estradiol ELISA Kit (Colorful Gene Biological Technology, Wuhan, China) according to the manufacturer’s protocol. Briefly, the samples were successively added to the micropores coated with mouse estradiol monoclonal antibody and then combined with horseradish peroxidase-labeled estradiol antibody to form antibody-antigen-enzyme labeled antibody complexes. After thorough washing, the substrate was added for color display. The concentration of estradiol in the sample was then calculated by measuring the absorbance at 450 nm using a microplate reader.

### Statistical analysis

The data are shown as mean ± SEM and statistically analyzed with a student’s *t*-test. *p* < 0.05 was considered a significant difference. All experiments were performed at least three times.

## Results

### ChIRP-MS identified 21 binding proteins of lncRNA Gm2044 in ovarian follicles

**Fig. 1:**
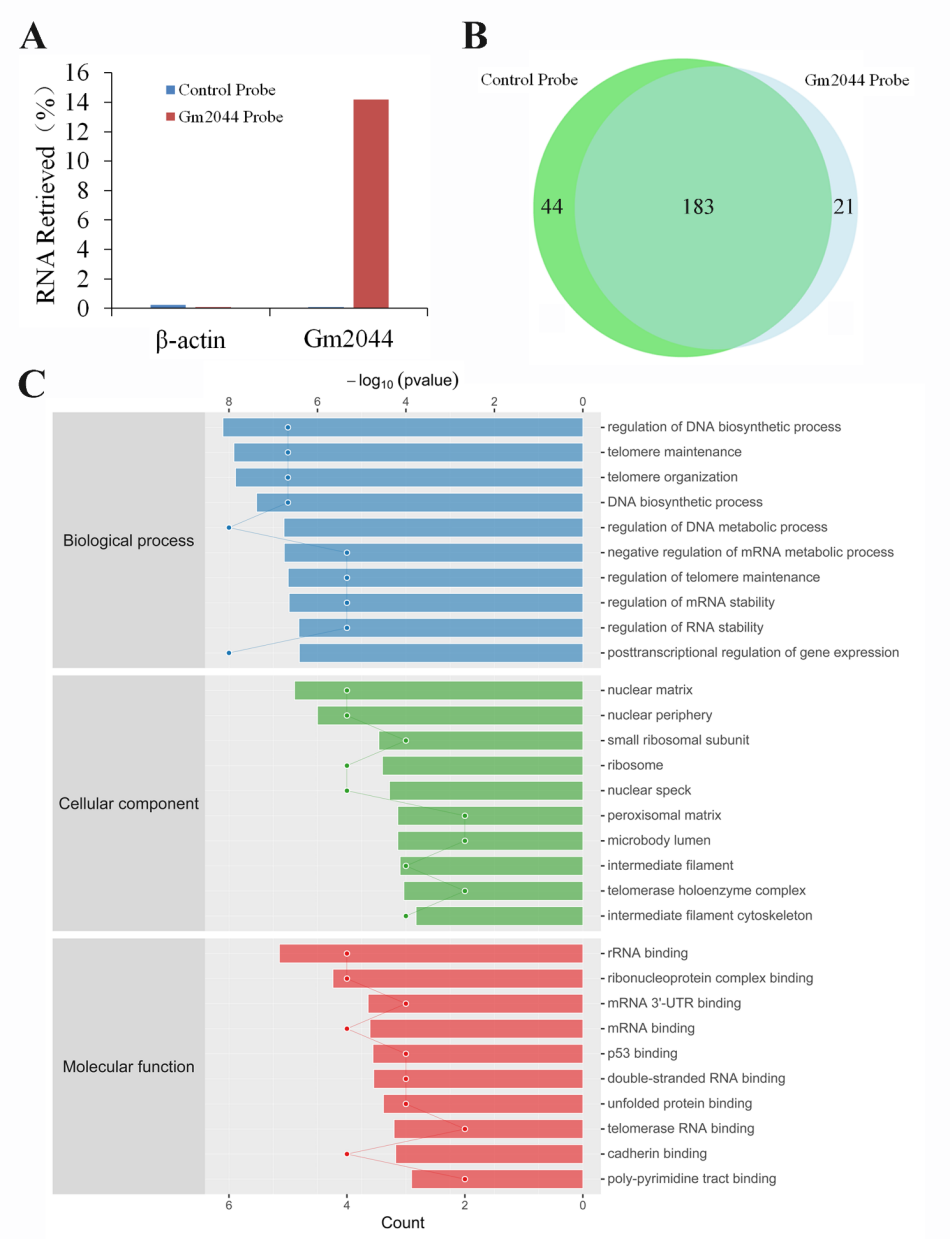
The specific proteins interacted with lncRNA Gm2044 and were subjected to GO analysis. **(A)** the lncRNA Gm2044 probe can successfully adsorb lncRNA Gm2044. The ovaries of adolescent mice were subjected to ChIRP using a control probe and Gm2044 probe, and the captured RNA was detected by qPCR with Gm2044 primers and β-Actin primers (negative control). **(B)** The lncRNA Gm2044 probe can specifically adsorb 21 proteins. The ovaries of adolescent mice were subjected to ChIRP-MS using a control probe and Gm2044 probe. **(C)** The specifically interacted proteins of lncRNA Gm2044 were analyzed by the GO method

To reveal the interacted protein of lncRNA Gm2044 and their function for these interactions in female reproduction, we isolated the ovaries of adolescent mice, which were subjected to ChIRP-MS analysis. The qPCR demonstrated that the probe of lncRNA Gm2044 could significantly capture more lncRNA Gm2044 than the control probe (Fig. 1A). Mass spectrometry showed that lncRNA Gm2044 captured 204 proteins and the control group captured 227 proteins, and 183 of them were the same proteins, which meant that lncRNA Gm2044 could specifically adsorb 21 proteins (Fig. 1B and Table S6).

**Fig. 2:**
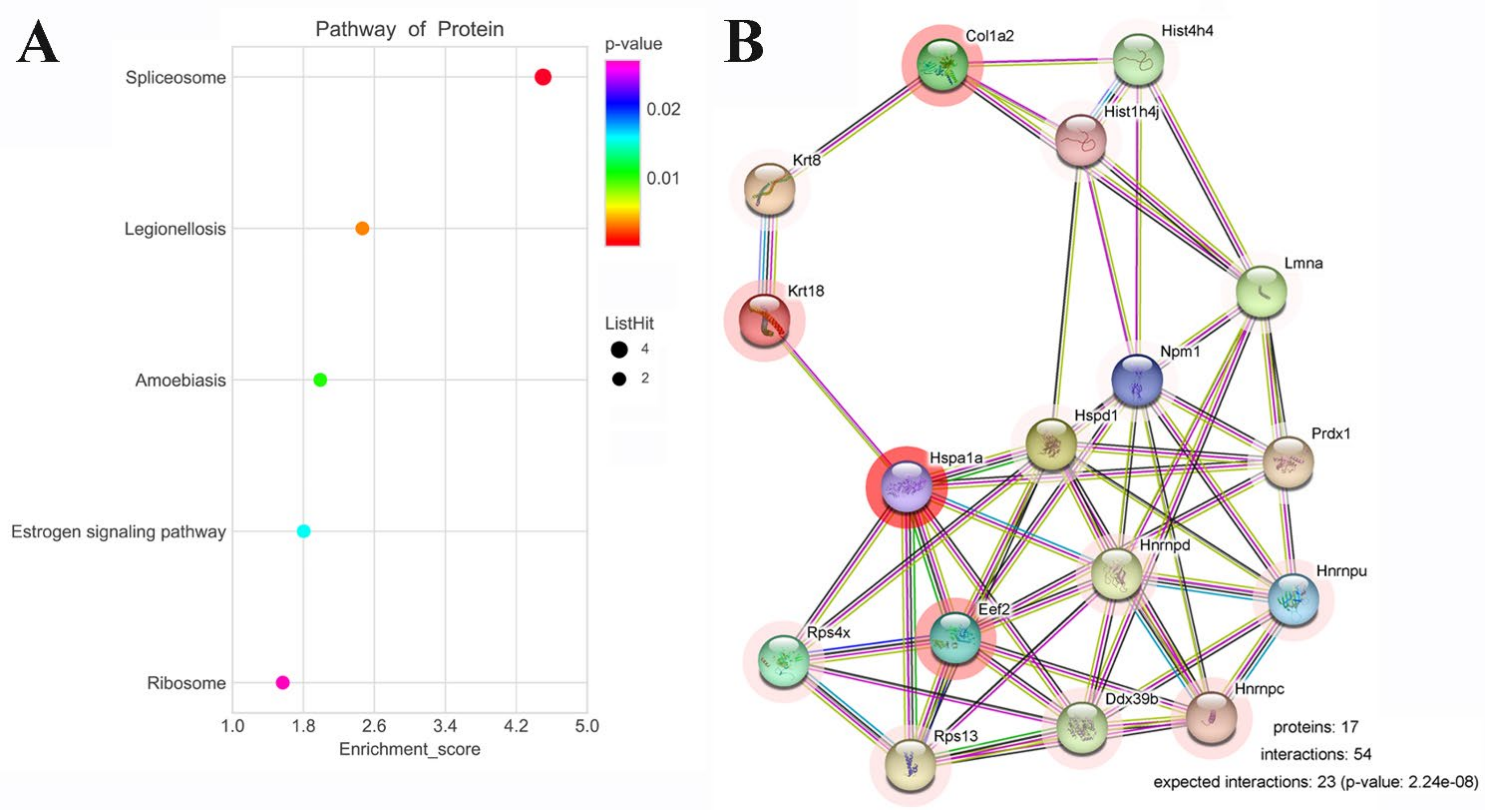
The specifically interacted proteins of lncRNA Gm2044 were analyzed by KEGG and STRING database. The estrogen signaling pathway and EEF2 interacting proteins were predicted by KEGG **(A)** and the STRING database **(B)**, respectively

GO was used to analyze the 21 binding proteins for lncRNA Gm2044 which revealed that posttranscriptional regulation of gene expression (GO: biological process), ribosome (GO: cellular component) and ribonucleoprotein complex binding (GO: molecular function), and EEF2 protein existed in these entries (Fig. 1C and Table S7). KEGG showed that the estrogen-signaling pathway involved the functions of lncRNA Gm2044 (Fig. 2A and Table S8). Based on these findings and our previous studies, we postulated that the EEF2, one of the binding proteins for lncRNA Gm2044, might mediate the functions of lncRNA Gm2044 regulating estradiol synthesis. Then, the STRING database analyzed the potential protein-protein interaction network for binding proteins of lncRNA Gm2044, which suggested that EEF2 and other proteins might co-mediate the function of lncRNA Gm2044 in female reproduction (Fig. 2B).

### LncRNA Gm2044 interacts with EEF2 protein

**Fig. 3:**
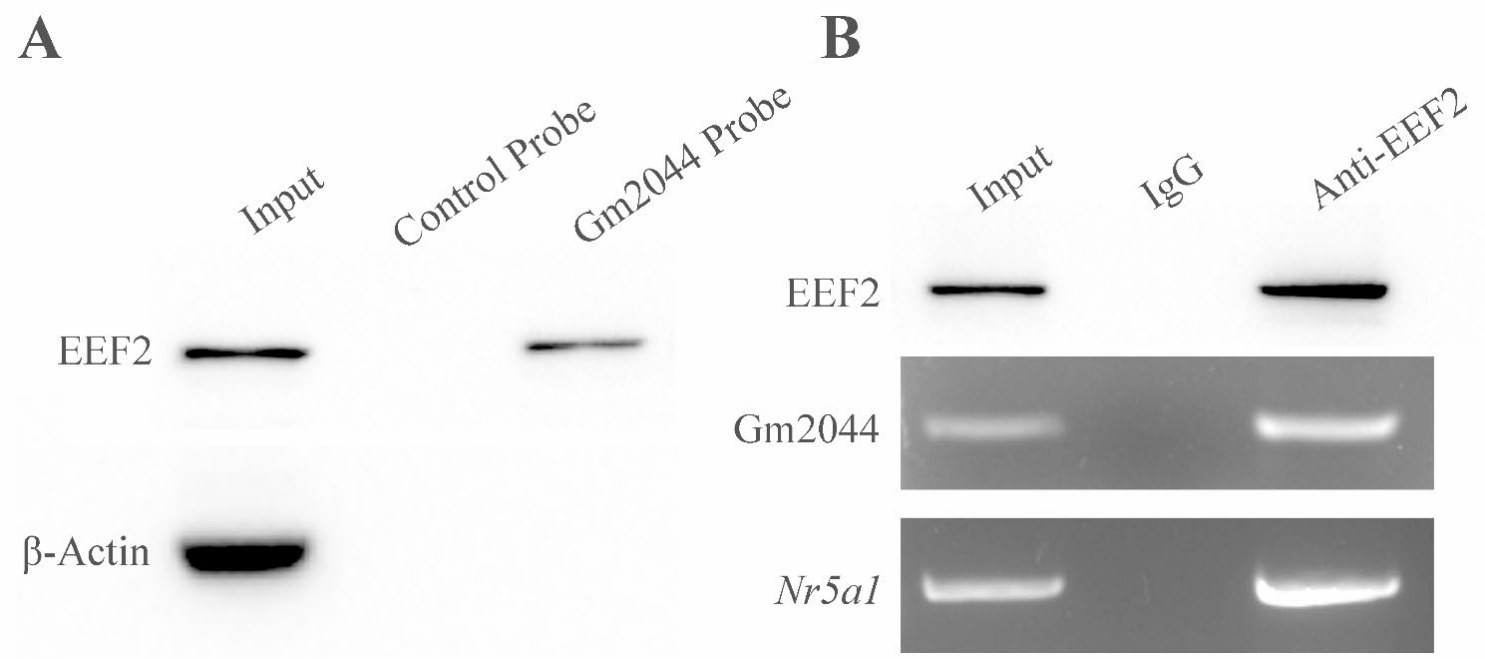
EEF2 protein interacted with lncRNA Gm2044. **(A)** The lncRNA Gm2044 can adsorb EEF2 protein. The control probe and Gm2044 probe were used to capture RNA-protein complex and then subjected to western blotting detecting EEF2 protein and β-Actin protein (negative control). **(B)** RNA IP revealed that EEF2 protein can capture lncRNA Gm2044 and bind *Nr5a1* mRNA. IgG and anti-EEF2 antibodies were used to adsorb protein-RNA complex and then subjected to western blotting, detecting EEF2 protein and RT-PCR detected lncRNA Gm2044 and *Nr5a1* mRNA.

ChIRP-MS and bioinformatics exploration for lncRNA Gm2044 showed that EEF2 might interact with lncRNA Gm2044, so we validated the potential interaction by molecular experiments. Western blotting experiments demonstrated that the lncRNA Gm2044 probe can adsorb EEF2 protein and the control probe was not capable of adsorbing EEF2 protein in the ovary, and both probes cannot adsorb negative control β-Actin (Fig. 3A). RNA IP, western blotting, and RT-PCR experiments verified that anti-EEF2 antibody can adsorb EEF2 protein and then pull the lncRNA Gm2044 and *Nr5a1* mRNA, and IgG did not adsorb EEF2 protein and did not pull the lncRNA Gm2044 and *Nr5a1* mRNA (Fig. 3B). These experiments confirmed that lncRNA Gm2044 can interact with EEF2 protein, which may regulate the function of lncRNA Gm2044.

### Estradiol synthesis was downregulated in lncRNA Gm2044 knockout mice

**Fig. 4:**
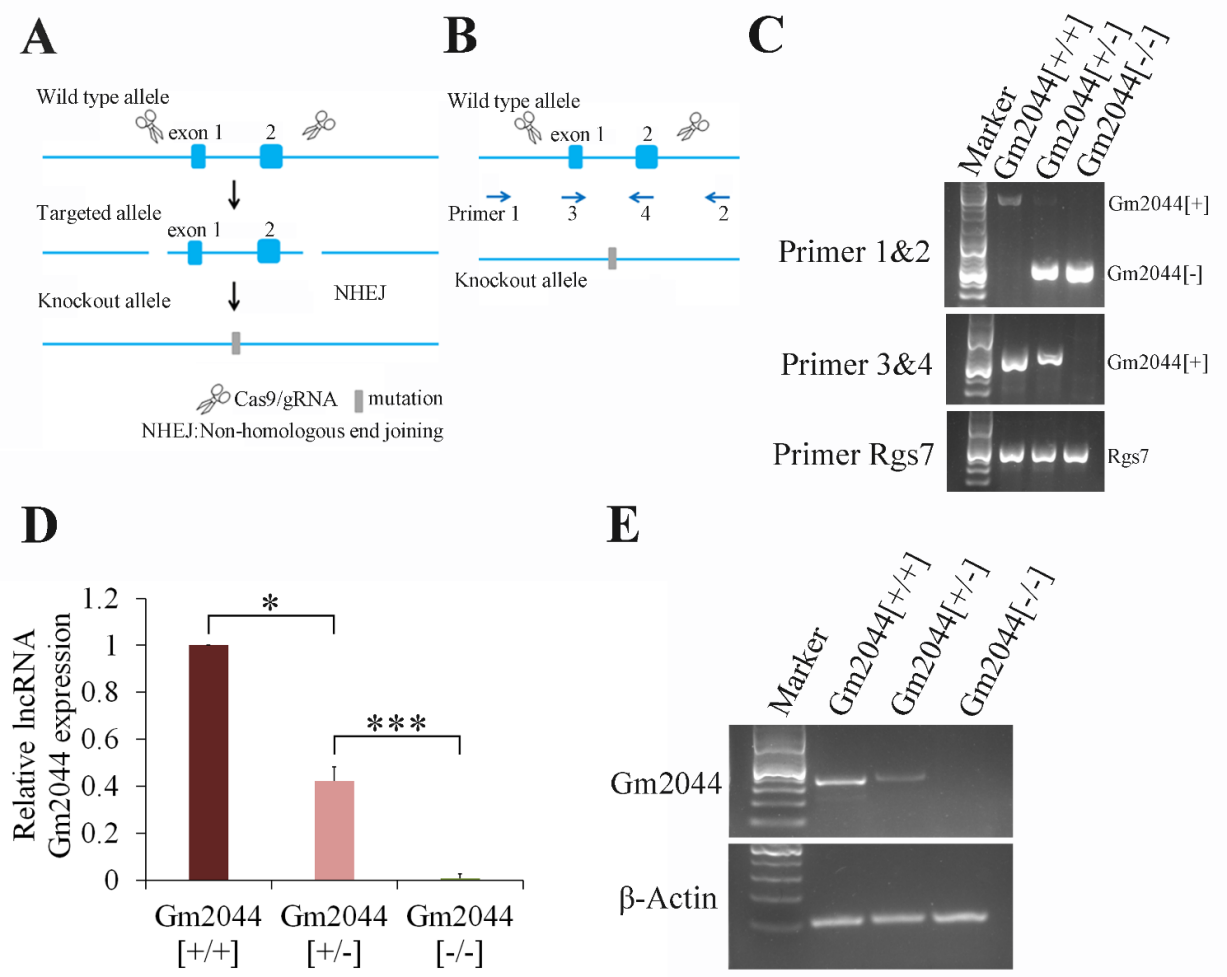
LncRNA Gm2044 knockout mice were successfully constructed. **(A)** The schematic diagram for exons 1 and 2 of lncRNA Gm2044 knockout using the CRISPR/Cas9 method. **(B)** The location of primers detecting genotype on lncRNA Gm2044 gene. **(C)** Genotyping of lncRNA Gm2044 for mice Genomic DNA was isolated from the mouse tail and then subjected to PCR using primer 1–4. Primer 1&2 got Gm2044 [+] fragment (2099 bp) and Gm2044 [-] fragment (531 bp). Primer 3&4 got Gm2044 [+] fragment (609 bp). *Rgs7* was used as the reference gene. **(D and E)** There was no expression of lncRNA Gm2044 in knockout mice. Total RNA was extracted from the ovary and then subjected to qPCR **(D)** and RT-PCR **(E)**. *, *p* < 0.05; ***, *p* < 0.001

For exploring the function of lncRNA Gm2044 function in vivo, we constructed the knockout mice of lncRNA Gm2044 whose exons 1 and 2 were specifically clipped using the CRISPR/Cas9 method (Fig. 4A). The genotyping assay revealed that the deficient mice lost exons 1 and 2 of lncRNA Gm2044 (Fig. 4B, C). The qPCR (Fig. 4D) and RT-PCR (Fig. 4E) demonstrated that the expression of lncRNA Gm2044 in the ovaries for Gm2044 [+/-] mice was about half that for Gm2044 [+/+] mice, and the ovaries of Gm2044 [-/-] mice did not express lncRNA Gm2044. These results revealed that we succeeded in obtaining knockout mice of lncRNA Gm2044.

**Fig. 5:**
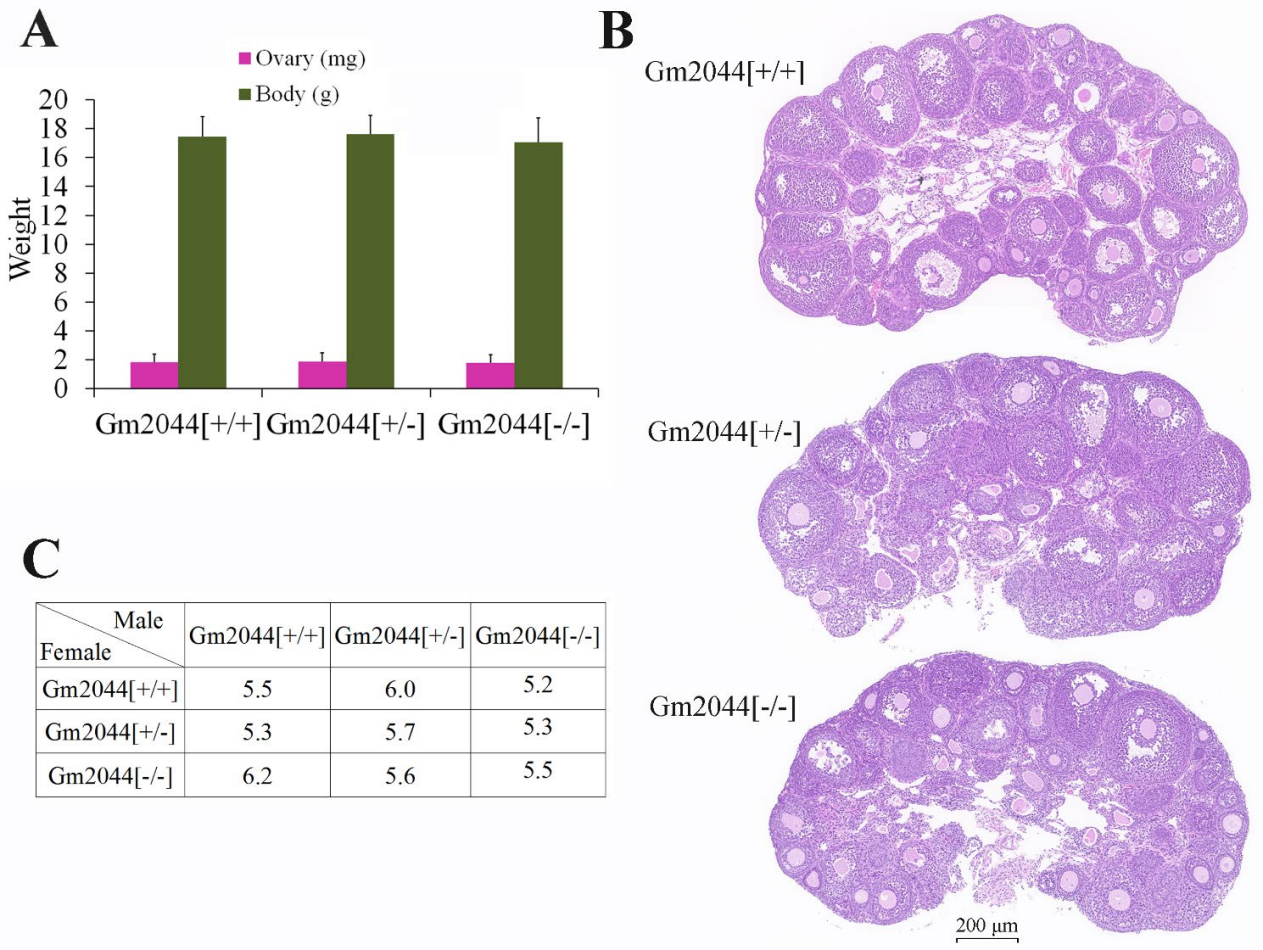
Reproductive phenotypes of lncRNA Gm2044 knockout mice were analyzed. **(A-C)** There were no differences in ovarian weight, follicular development, and fertility between Gm2044 [+/+] mice and Gm2044 [-/-] mice. The weights of the ovary and body were statistically analyzed **(A)** and ovaries were detected by H&E analysis **(B)**. The mating experiment of Gm2044[+/+], Gm2044 [+/-] and Gm2044 [-/-] mice were also executed **(C)**

Next, the reproductive phenotypes of lncRNA Gm2044 knockout mice were determined by statistics and molecular experiments. The weights of the ovaries and mouse bodies for Gm2044 [-/-] mice had no significant difference compared with Gm2044 [+/+] and Gm2044 [+/-] mice (Fig. 5A). H&E detection showed that the follicular development in Gm2044 [-/-] mice was normal compared with that in Gm2044 [+/+] and Gm2044 [+/-] mice (Fig. 5B). The mouse mating experiment also revealed that the fertility of Gm2044 [-/-] mice was similar to Gm2044 [+/+] and Gm2044 [+/-] mice in males and females (Fig. 5C).

**Fig. 6:**
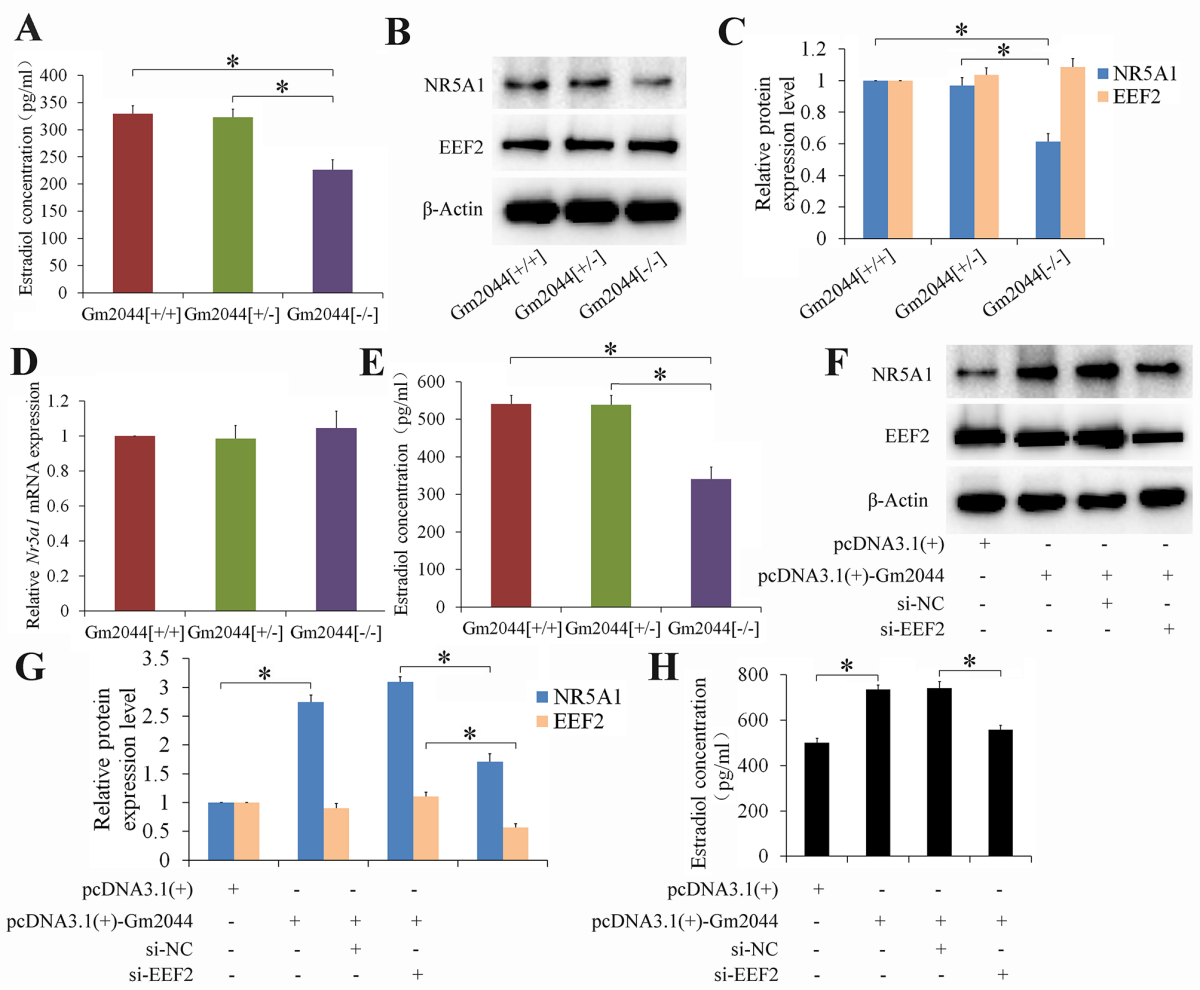
Estradiol concentration significantly decreased in female Gm2044 [-/-] mice. **(A)** The serum estradiol concentration of female lncRNA Gm2044 knockout mice significantly decreased. Serums were isolated from Gm2044[+/+], Gm2044 [+/-] mice and Gm2044 [-/-] mice and then were used to analyze estradiol concentration by ELISA method. **(B and C)** The NR5A1 protein expression significantly decreased in follicular granulosa cells of Gm2044 [-/-] mice. The protein for follicular granulosa cells of Gm2044[+/+], Gm2044 [+/-] mice and Gm2044 [-/-] mice were isolated and then subjected to western blotting (B) and quantitative analysis (C). **(D)** The *Nr5a1* mRNA expression had no change in follicular granulosa cells of Gm2044 [-/-] mice compared with that in Gm2044 [+/+] mice. The RNA for follicular granulosa cells of Gm2044[+/+], Gm2044 [+/-] mice and Gm2044 [-/-] mice were isolated and then subjected to qPCR. **(E)** The estradiol level for follicular granulosa cells of Gm2044 [-/-] mice significantly decreased. Culture medium for follicular granulosa cells of Gm2044[+/+], Gm2044 [+/-] mice and Gm2044 [-/-] mice were used to analyze estradiol concentration by ELISA method. **(F and G)** Knockdown of EEF2 can reverse the elevated effects of Gm2044 on NR5A1 protein level in follicular granulosa cells of Gm2044 [-/-] mice. The protein was isolated from Gm2044 [-/-] mouse follicular granulosa cells transfected with indicated plasmid and siRNA, and then subjected to western blotting (F) and quantitative analysis (G). **(H)** Knockdown of EEF2 can reverse the elevated effects of Gm2044 on estradiol concentration in follicular granulosa cells of Gm2044 [-/-] mice. Culture medium for Gm2044 [-/-] mouse follicular granulosa cells transfected with indicated plasmid and siRNA were used to analyze estradiol concentration by ELISA method. si-EEF2, siRNA for EEF2 () and siRNA for negatice control (si-NC) *, *p* < 0.05

Our previous research demonstrated that lncRNA Gm2044 could promote estradiol synthesis in follicular granulosa cells [20]. The serum estradiol concentration of female lncRNA Gm2044 knockout mice was assayed using the ELISA method, which revealed that the serum estradiol concentration in female Gm2044 [-/-] mice was significantly decreased compared with that in female Gm2044 [+/+] and Gm2044 [+/-] mice (Fig. 6A). The key transcriptional factor *Nr5a1* for estradiol synthesis was analyzed, and results showed that NR5A1 protein expression was significantly reduced and EEF2 protein expression had no significant difference in follicular granulosa cells of Gm2044 [-/-] mice compared with that of Gm2044 [+/+] and Gm2044 [+/-] mice (Fig. 6B, C). However, the *Nr5a1* mRNA had no significant change in follicular granulosa cells of Gm2044 [-/-] mice compared with that of Gm2044 [+/+] and Gm2044 [+/-] mice (Fig. 6D). Furthermore, the estradiol concentration in follicular granulosa cells of Gm2044 [-/-] mice significantly decreased compared with that in Gm2044 [+/+] and Gm2044 [+/-] mice (Fig. 6E). Furthermore, overexpression of Gm2044 promoted NR5A1 protein level and estradiol concentration in follicular granulosa cells of Gm2044 [-/-] mice, and knockdown of EEF2 can reverse the elevated effects of Gm2044 on NR5A1 protein level and estradiol concentration (Fig. 6F, H). These results suggested that lncRNA Gm2044 can interact with translational regulatory protein EEF2, and they may promote the binding of EEF2 to *Nr5a1* mRNA and enhance the *Nr5a1* mRNA translation. In addition, the upregulated NR5A1 protein can strengthen estradiol synthesis, which needs to be further studied.

### Transcriptome analysis in ovaries of adult lncRNA Gm2044 knockout mice

**Fig. 7:**
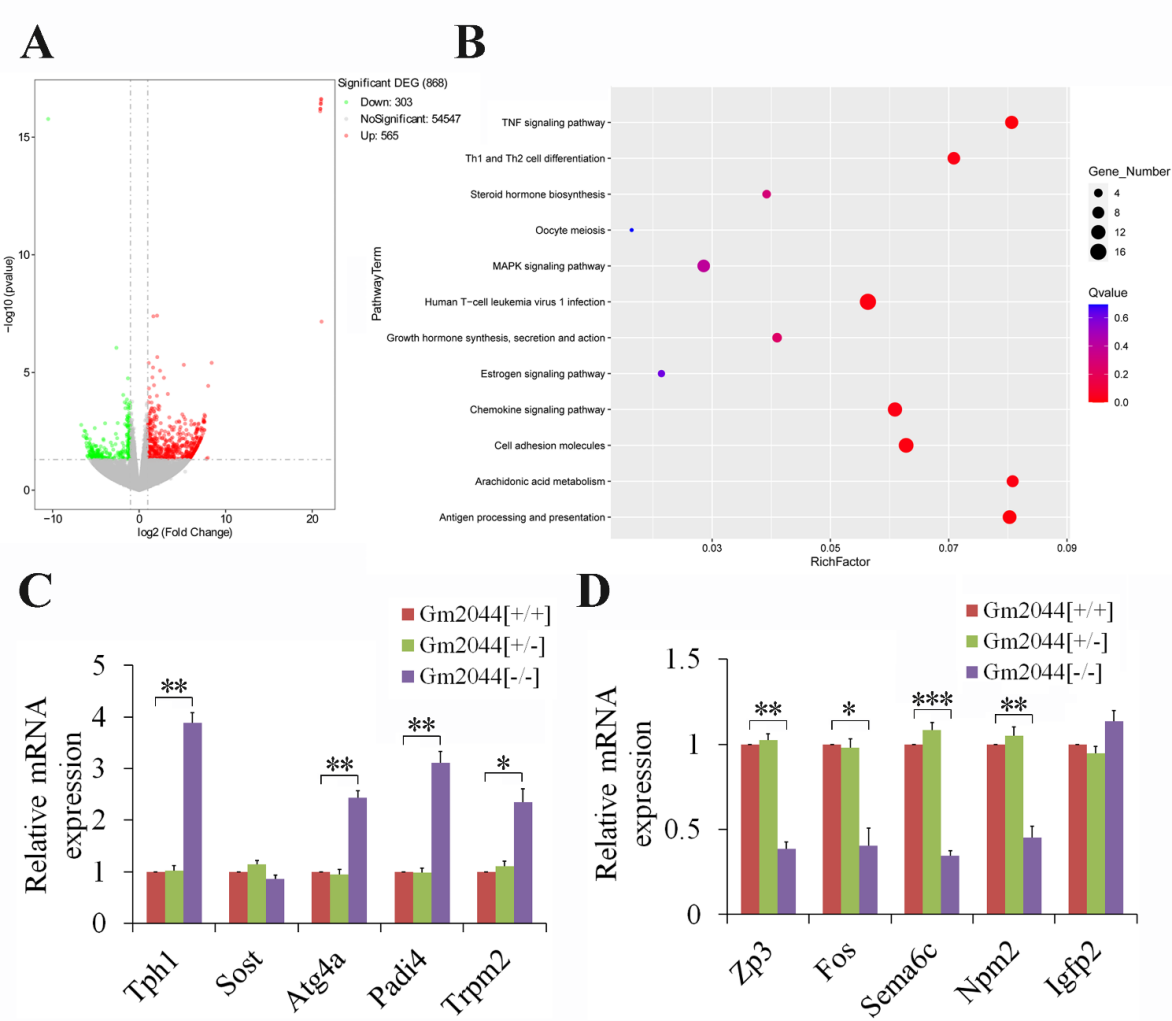
Transcriptome study in ovaries of Gm2044 [+/-] and Gm2044 [-/-] mice. **(A and B)** Volcano plot **(A)** and KEGG **(B)** comparing differentially expressed genes for Gm2044 [-/-] and Gm2044 [+/-] mice. **(C and D)** Upregulated genes **(C)** and downregulated genes **(D)** for transcriptome were validated by qPCR. Total RNA was isolated from ovaries of Gm2044 [+/+], Gm2044 [+/-] and Gm2044 [-/-] mice and then used to detect relative mRNA expression by qPCR. *, *p* < 0.05; **, *p* < 0.01; ***, *p* < 0.001

To determine the potential signaling pathway of the lncRNA Gm2044 regulation of estradiol synthesis, transcriptome sequencing was performed for ovaries of adult lncRNA Gm2044 knockout mice, which identified 565 significant up-regulated genes and 303 significant down-regulated genes (Fig. 7A, Figure S1 and Table S9). Differentially expressed genes were then analyzed with GO (Figure S2) and KEGG (Fig. 7B), which revealed some ovarian development and estrogen-related pathways, such as endoribonuclease activity (GO: molecular function), integral component of membrane (GO: cellular component), positive regulation of ovarian follicle development (GO: biological process), and estrogen signaling pathway (KEGG). Some of the differentially expressed genes were related to ovarian follicle development or estradiol synthesis, such as upregulated genes (*Tph1*, *Sost*, *A tg4a*, *Padi4*, and *Trpm2*) and downregulated genes (*Zp3*, *Fos*, *Sema6c*, *Npm2*, and *Igfp2*) which were then validated by qPCR (Fig. 7C and D). The expression trend of the above genes was consistent between transcriptome sequencing and qPCR except for *Sost* and *Igfp2* (Fig. 7C and D). Understanding the lncRNA Gm2044/EEF2 protein regulation of estradiol synthesis will provide new knowledge for lncRNA study in female reproduction.

## Discussion

Our research group revealed that lncRNA Gm2044 is highly expressed in the mouse ovary and testis [16]. In testis, lncRNA Gm2044 interacted with *Utf1* mRNA to inhibit UTF1 protein expression and served as a miRNA-202 host gene to regulate *Rbfox2* expression, which then affected male germ cell function [16, 22]. Furthermore, transcription factor A-MYB can promote lncRNA Gm2044 expression, which modifies the spermatocyte cell line GC-2 function through the miRNA-335-3p/Sycp1 signaling pathway [18]. In the ovary, lncRNA Gm2044 can serve as a miRNA-138-5p sponge and then enhance estradiol levels in mouse preantral granulosa cells [20]. To explore the function of lncRNA Gm2044 in regulating female reproduction in vivo, we used the CRISPR/Cas9 method to acquire lncRNA Gm2044 knockout mice and the ChIRP-MS method to reveal binding proteins of lncRNA Gm2044 in the ovary.

Recent studies have highlighted the significance of the EEF2 protein in the occurrence of diseases [23–25]. Blocking the interaction of EEF2 and lactate dehydrogenase A can release EEF2, which facilitates megakaryocyte maturation and platelet production [24]. The heterozygous missense variant of *Eef2* lead to benign external hydrocephalus through impairing nervous system development [26]. High expression of lncRNA MALAT1 in non-small cell lung cancer accelerated tumorigenesis via adsorbing miRNA-515-5p and then upregulating EEF2 protein levels [27]. The downregulation of EEF2 protein mediated the lipopolysaccharide-induced acute pulmonary inflammation on microvascular endothelial cells [28]. This study proved that lncRNA Gm2044 can interact with EEF2 protein and then promote estradiol synthesis. Knockdown of EEF2 can reverse the elevated effects of Gm2044 on NR5A1 protein level and estradiol concentration in follicular granulosa cells of Gm2044 [-/-] mice. The potential molecular mechanism may be the interaction of lncRNA Gm2044 and EEF2 facilitating the binding of EEF2 to *Nr5a1* mRNA and then enhancing the *Nr5a1* mRNA translation, which stimulates the expression of estrogen synthase. The specific mechanism of lncRNA Gm2044 and EEF2 regulating estradiol synthesis needs to be further studied.

LncRNAs have been reported to play important roles in reproduction and some lncRNAs have been explored by knockout mouse models and antisense oligonucleotide systems in vivo [29–31]. Antisense oligonucleotides of lncRNA Tsx were microinjected into seminiferous tubules and then promoted germ cell apoptosis through inhibiting lncRNA Tsx expression [29]. Knockout of lncRNA THOR in male mice caused a significant decrease in survival rate, body weight, testis weight, spermatozoa motility and fertility, and downregulation of the MEK-ERK signaling pathway [32]. Depletion of lncRNA Tug1 locus resulted in abnormal spermatozoa number and morphology which induced male infertility [30]. LncRNA Neat1 is highly expressed in the corpus luteum and the deletion of lncRNA Neat1 induced partial defects of corpus luteum and stochastic female infertility [31].

To study lncRNA Gm2044 in vivo, the CRISPR/Cas9 method was used to construct the lncRNA Gm2044 knockout mice. The follicular development and fertility of lncRNA-Gm2044-deficient female mice were not affected, but the serum estradiol concentration in lncRNA-Gm2044-deficient female mice significantly decreased. Furthermore, transcriptome sequencing was executed for ovaries of adult lncRNA Gm2044 deficient mice. Some of the differentially expressed genes were related to estradiol syntheses, such as upregulated genes (*Tph1*, *Sost*, *A tg4a*, *Padi4*, and *Trpm2*) and downregulated genes (*Zp3*, *Fos*, *Sema6c*, *Npm2*, and *Igfp2*). GO and KEGG revealed some estrogen-related pathways, such as endoribonuclease activity (GO: molecular function), an integral component of membrane (GO: cellular component), positive regulation of ovarian follicle development (GO: biological process), and estrogen signaling pathway (KEGG). We will investigate whether low estradiol concentrations affect the reproductive systems of middle-aged and elderly mice in the future.

In this research, we identified 21 binding proteins of lncRNA Gm2044 in ovarian follicles. Molecular experiments confirmed that lncRNA Gm2044 can interact with EEF2 protein. The analysis of lncRNA Gm2044 knockout mice showed that the follicular development and fertility in lncRNA-Gm2044-deficient female mice were not affected. However, the serum estradiol concentration in lncRNA Gm2044 knockout female mice significantly decreased. In addition, transcriptome sequencing identified 565 significant up-regulated genes and 303 significant down-regulated genes in the ovaries of lncRNA-Gm2044-deficient mice. Differentially expressed genes were then analyzed with GO and KEGG, which revealed some estrogen-related pathways and provided a new idea for the treatment of female infertility.

### Electronic supplementary material

Below is the link to the electronic supplementary material.

**Supplementary Material 1**

**Supplementary Material 2**

**Supplementary Material 3**

**Supplementary Material 4**

**Supplementary Material 5**

**Supplementary Material 6**

**Supplementary Material 7**

## Data Availability

The data for this research can be acquired from the corresponding author.

## References

[1] Qin L (2022). Expression of lncRNA TINCR in the placenta of patients with pre-eclampsia and its effect on the biological behaviours of trophoblasts. Zygote.

[2] Zhang S (2021). Silencing of AFAP1-AS1 lncRNA impairs cell proliferation and migration by epigenetically promoting DUSP5 expression in pre-eclampsia. J Cell Biochem.

[3] Wang MM, Zhong JX, Xiang YY (2021). LncRNA-GAS5 related to the processes of recurrent pregnancy loss by regulating Th1/Th2 balance. Kaohsiung J Med Sci.

[4] Zhao H (2021). LncRNA LINC01088 inhibits the function of trophoblast cells, activates the MAPK-signaling pathway and associates with recurrent pregnancy loss. Mol Hum Reprod.

[5] Zhen J (2021). Downregulating lncRNA NEAT1 induces proliferation and represses apoptosis of ovarian granulosa cells in polycystic ovary syndrome via microRNA-381/IGF1 axis. J Biomed Sci.

[6] Zheng W (2022). cAMP-response element binding protein mediates podocyte injury in diabetic nephropathy by targeting lncRNA DLX6-AS1. Metabolism.

[7] Shi M (2021). LncRNA-SNHG16 promotes proliferation and migration of acute myeloid leukemia cells via PTEN/PI3K/AKT axis through suppressing CELF2 protein. J Biosci.

[8] Xu A (2020). LncRNA H19 suppresses osteosarcomagenesis by regulating snoRNAs and DNA repair protein complexes. Front Genet.

[9] Wang H (2022). The lncRNA ZFAS1 regulates lipogenesis in colorectal cancer by binding polyadenylate-binding protein 2 to stabilize SREBP1 mRNA. Mol Ther Nucleic Acids.

[10] Priyanka P (2021). The lncRNA HMS recruits RNA-binding protein HuR to stabilize the 3’-UTR of HOXC10 mRNA. J Biol Chem.

[11] Sun L, Zhang P, Lu W. lncRNA MALAT1 Regulates Mouse Granulosa Cell Apoptosis and 17beta-Estradiol Synthesis via Regulating miR-205/CREB1 Axis. Biomed Res Int, 2021. 2021: p. 6671814. 10.1155/2021/6671814 PMC7904346 33681369

[12] Han DX (2019). Differentially expressed lncRNA-m433s1 regulates FSH secretion by functioning as a miRNA sponge in male rat anterior pituitary cellsdagger. Biol Reprod.

[13] Sun H (2015). H19 lncRNA mediates 17beta-estradiol-induced cell proliferation in MCF-7 breast cancer cells. Oncol Rep.

[14] Yin DD (2015). Downregulation of lncRNA TUG1 affects apoptosis and insulin secretion in mouse pancreatic beta cells. Cell Physiol Biochem.

[15] Bhan A (2013). Antisense transcript long noncoding RNA (lncRNA) HOTAIR is transcriptionally induced by estradiol. J Mol Biol.

[16] Hu K (2018). Gm2044 highly expresses in spermatocyte and inhibits Utf1 translation by interacting with Utf1 mRNA. Genes Genomics.

[17] Chu C (2015). Systematic discovery of Xist RNA binding proteins. Cell.

[18] Liang M (2020). LncRNA-Gm2044 is transcriptionally activated by A-MYB and regulates Sycp1 expression as a mir-335-3p sponge in mouse spermatocyte-derived GC-2spd(ts) cells. Differentiation.

[19] Liang WC (2015). The lncRNA H19 promotes epithelial to mesenchymal transition by functioning as miRNA sponges in colorectal cancer. Oncotarget.

[20] Hu K (2019). Gm2044 promotes 17beta-estradiol synthesis in mpGCs by acting as mir-138-5p sponge. Mol Reprod Dev.

[21] Wang K et al. Is Parthanatos involved in Varicocele? DNA cell Biol, 2022. 41(10): p. 861–70. 10.1089/dna.2022.0289 36067068

[22] Liang M (2019). Upregulated lncRNA Gm2044 inhibits male germ cell development by acting as miR-202 host gene. Anim Cells Syst (Seoul).

[23] Kasica NP (2022). Antagonists targeting eEF2 kinase rescue multiple aspects of pathophysiology in Alzheimer’s disease model mice. J Neurochem.

[24] Chen Q (2022). Inhibition of LDHA to Induce EEF2 Release enhances Thrombocytopoiesis. Blood.

[25] Tsuda-Sakurai K, Kimura M, Miura M (2020). Diphthamide modification of eEF2 is required for gut tumor-like hyperplasia induced by oncogenic ras. Genes Cells.

[26] Nabais Sa MJ (2021). De Novo variants in EEF2 cause a neurodevelopmental disorder with benign external hydrocephalus. Hum Mol Genet.

[27] Rong F (2020). MALAT1 promotes cell tumorigenicity through regulating miR-515-5p/EEF2 Axis in Non-Small Cell Lung Cancer. Cancer Manag Res.

[28] Fei L, Sun G, You Q (2020). miR-642a-5p partially mediates the effects of lipopolysaccharide on human pulmonary microvascular endothelial cells via eEF2. FEBS Open Bio.

[29] Chen Z (2021). Microinjection of antisense oligonucleotides into living mouse testis enables lncRNA function study. Cell Biosci.

[30] Lewandowski JP (2020). The Tug1 lncRNA locus is essential for male fertility. Genome Biol.

[31] Nakagawa S (2014). The lncRNA Neat1 is required for corpus luteum formation and the establishment of pregnancy in a subpopulation of mice. Development.

[32] Zhou L (2021). Investigation of the lncRNA THOR in mice highlights the importance of noncoding RNAs in mammalian Male Reproduction. Biomedicines.

