# Correction: Interaction of lncRNA Gm2044 and EEF2 promotes estradiol synthesis in ovarian follicular granulosa cells

**DOI:** 10.1186/s13048-023-01287-y

**Published:** 2023-10-06

**Authors:** Ke Hu, Chen Wang, Yifan Xu, Fan Li, Xuefeng Han, Chuanwang Song, Meng Liang

**Affiliations:** 1 School of Life Science, Bengbu Medical College, Bengbu, China; 2 School of Laboratory Medicine, Bengbu Medical College, Bengbu, China

**Correction: J Ovarian Res 16:171 (2023)**

10.1186/s13048-023-01232-z

Following publication of the original article [1], the authors found two mistakes while reading the article today. In Fig. 6A and E as follow, the names of the first and second column should be Gm2044[+/+] and Gm2044[+/-], respectively.

The correct figure is shown here and the original article has been corrected.

**Fig. 6:**
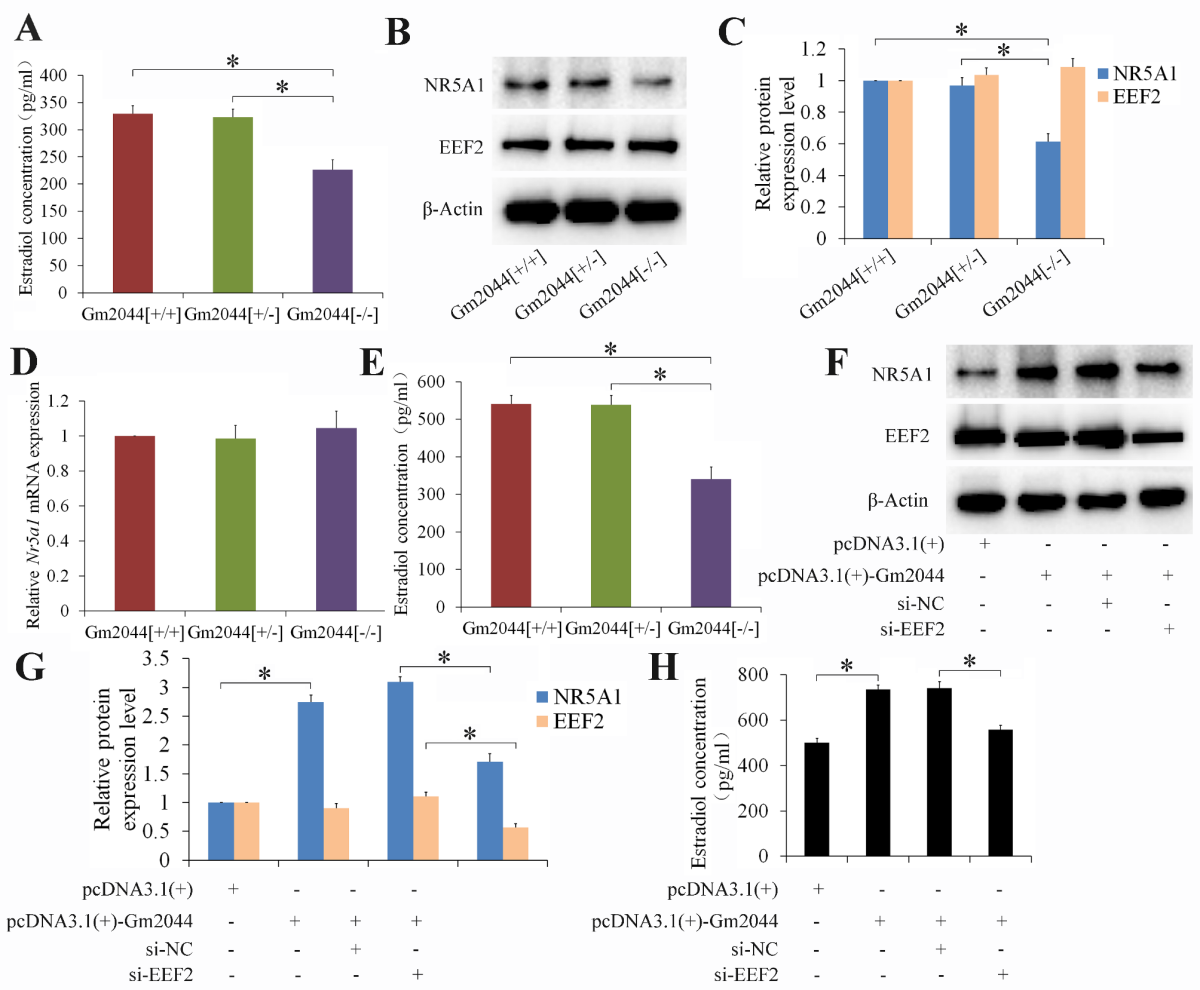
Estradiol concentration significantly decreased in female Gm2044 [−/−] mice. (**A**) The serum estradiol concentration of female lncRNA Gm2044 knockout mice significantly decreased. Serums were isolated from Gm2044[+/+], Gm2044 [+/−] mice and Gm2044 [−/−] mice and then were used to analyze estradiol concentration by ELISA method. (**B and C**) The NR5A1 protein expression significantly decreased in follicular granulosa cells of Gm2044 [−/−] mice. The protein for follicular granulosa cells of Gm2044[+/+], Gm2044 [+/−] mice and Gm2044 [−/−] mice were isolated and then subjected to western blotting (B) and quantitative analysis (C). (**D**) The Nr5a1 mRNA expression had no change in follicular granulosa cells of Gm2044 [−/−] mice compared with that in Gm2044 [+/+] mice. The RNA for follicular granulosa cells of Gm2044[+/+], Gm2044 [+/−] mice and Gm2044 [−/−] mice were isolated and then subjected to qPCR. (**E**) The estradiol level for follicular granulosa cells of Gm2044 [−/−] mice significantly decreased. Culture medium for follicular granulosa cells of Gm2044[+/+], Gm2044 [+/−] mice and Gm2044 [−/−] mice were used to analyze estradiol concentration by ELISA method. (**F and G**) Knockdown of EEF2 can reverse the elevated effects of Gm2044 on NR5A1 protein level in follicular granulosa cells of Gm2044 [−/−] mice. The protein was isolated from Gm2044 [−/−] mouse follicular granulosa cells transfected with indicated plasmid and siRNA, and then subjected to western blotting (F) and quantitative analysis (G). (**H**) Knockdown of EEF2 can reverse the elevated effects of Gm2044 on estradiol concentration in follicular granulosa cells of Gm2044 [−/−] mice. Culture medium for Gm2044 [−/−] mouse follicular granulosa cells transfected with indicated plasmid and siRNA were used to analyze estradiol concentration by ELISA method. si-EEF2, siRNA for EEF2 () and siRNA for negatice control (si-NC) *, *p* < 0.05
