# Supplementary material for: Interaction of lncRNA Gm2044 and EEF2 promotes estradiol synthesis in ovarian follicular granulosa cells
Source: J Ovarian Res. 2023 Aug 23;16:171. doi: 10.1186/s13048-023-01232-z (PMC10464411; doi:10.1186/s13048-023-01232-z)
Supplement: Supplementary file 1 — Supplementary Material 1 [file 13048_2023_1232_MOESM1_ESM.docx]

**Figure S1.** Heatmap comparing differentially expressed genes for Gm2044 [+/-] and Gm2044 [-/-] mice.


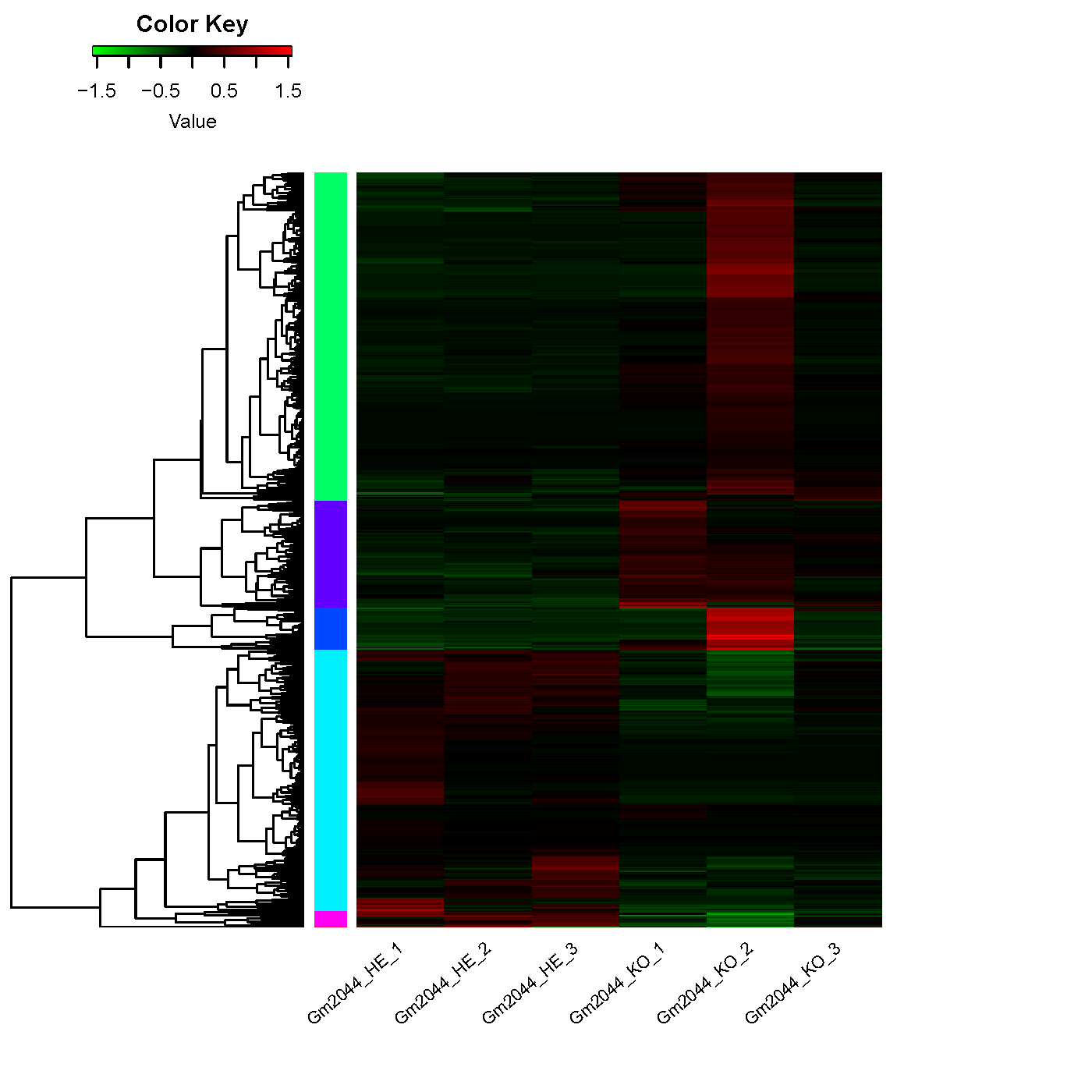


**Figure S2.** GO comparing differentially expressed genes for Gm2044 [+/-] and Gm2044 [-/-] mice.

**
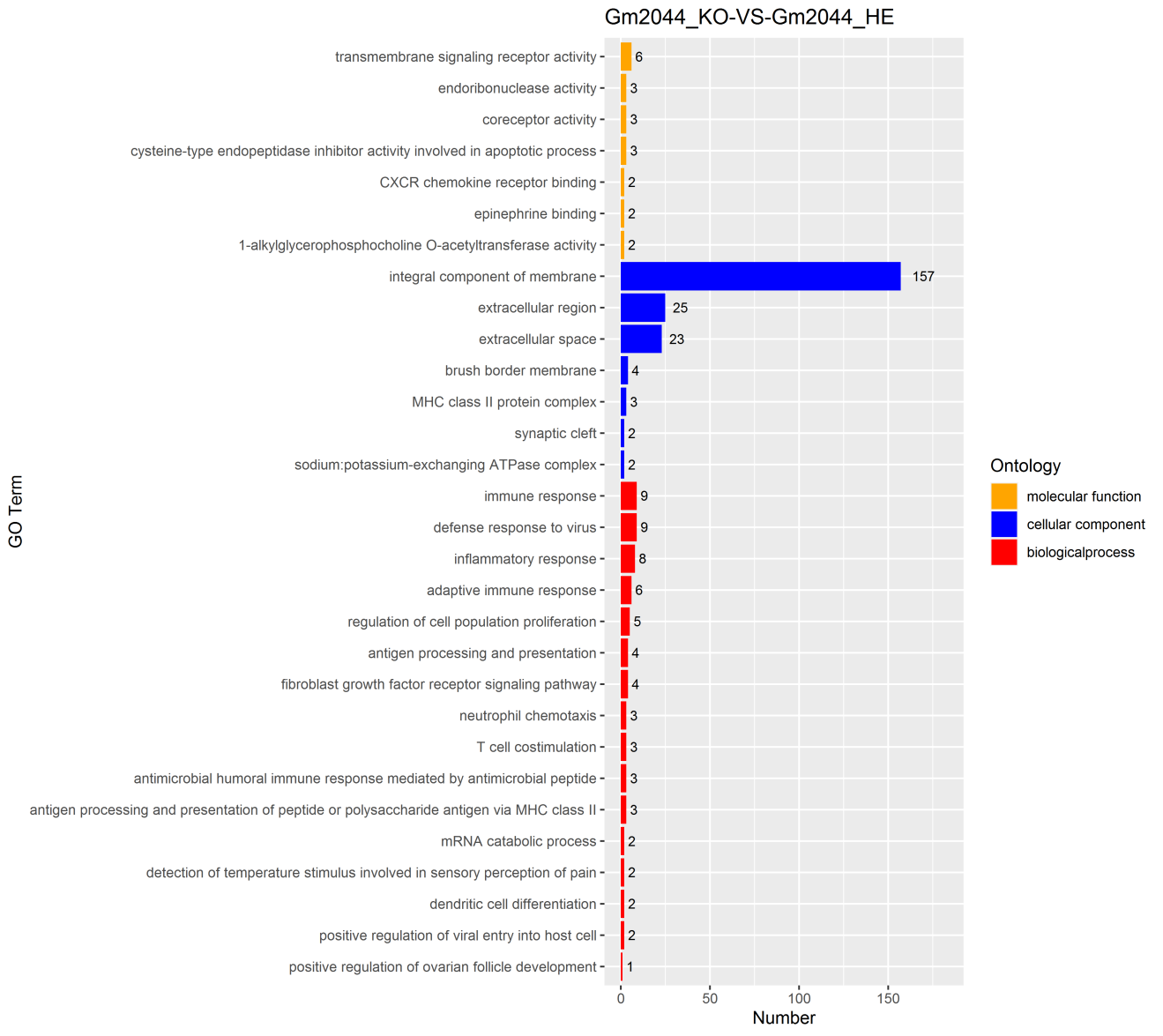
**
