# Supplementary material for: Interaction of lncRNA Gm2044 and EEF2 promotes estradiol synthesis in ovarian follicular granulosa cells
Source: J Ovarian Res. 2023 Aug 23;16:171. doi: 10.1186/s13048-023-01232-z (PMC10464411; doi:10.1186/s13048-023-01232-z)
Supplement: Supplementary file 2 — Supplementary Material 2 [file 13048_2023_1232_MOESM2_ESM.docx]

**Table S1.** Sequences of gRNAs for lncRNA Gm2044 knockout mice.

**gRNA number sequence (5’-3’)**

gRNA 1 GGCCAGCAGCCCCCTACCCTGGG

gRNA 2 CATGTGGGTATTACAAGGGAAGG

**Table S2.** List of primer pairs for genotyping assay.

**Gene Forward primer sequence (5’-3’) Reverse primer sequence (5’-3’)**

Gm2044 Primer 1&2 ATGCCCCAATGGGAGTTCTG CATCCCCAGGGAAGGTCCTA

Gm2044 Primer 3&4 CAGAACACCCTCCATGGGTC AGAGGACTTGGCTGTTGCTC

**Table S3.** List of probes for ChIRP.

**Probe number Probe sequence (5’-3’)**

Gm2044 Probe 1 TTGGCTGTTGCTCGGTGAAG

Gm2044 Probe 2 AGCAGTCACCAGAAGGATTG

Gm2044 Probe 3 CTATACCTCTTTAGACCAGA

Gm2044 Probe 4 GTGGAAGGGAAGCAGAAGTC

Gm2044 Probe 5 CTTGCGTAGGAATCACTGTT

Gm2044 Probe 6 AACCTCGGATAGGTCAGATG

Gm2044 Probe 7 TCTTCTAAGGTCCCTAGAAC

Gm2044 Probe 8 CGTCATCCACTAGTGTGAAA

Gm2044 Probe 9 TGTAATCATGGTGCGAAGCA

**Table S4.** List of primer pairs for qPCR.

**Gene Forward primer sequence (5’-3’) Reverse primer sequence (5’-3’)**

Gm2044 TAGCGCATGGGAAGATGGAG AGGCCATCTCTTGCTCAGAC

**Table S5.** List of primer pairs for RT-PCR.

**Gene Forward primer sequence (5’-3’) Reverse primer sequence (5’-3’)**

Gm2044 TTCTCAGGCCACTAGCAGTC AGGCCATCTCTTGCTCAGAC

*Nr5a1*  CCCGAGTGGCCGTCATTGCG CTTGAAGAAGCCCTTGCAGC
